# Early pregnancy loss incidence in high-income settings: a protocol for a systematic review and meta-analysis

**DOI:** 10.1186/s13643-021-01815-1

**Published:** 2021-10-25

**Authors:** L. Schummers, N. Oveisi, M. S. Ohtsuka, J. A. Hutcheon, K. A. Ahrens, J. Liauw, W. V. Norman

**Affiliations:** 1 Department of Family Practice, University of British Columbia, E303 - 4500 Oak Street, Vancouver, BC V6H 3N1 Canada; 2 School of Population and Public Health, University of British Columbia, 2206 E Mall, Vancouver, BC V6T 1Z3 Canada; 3 Department of Obstetrics and Gynaecology, University of British Columbia, Suite 930, 1125 Howe Street, Vancouver, BC V6Z 2K8 Canada; 4 Muskie School of Public Service, University of Southern Maine, 32 Bedford St, Portland, ME 04101 USA; 5 Faculty of Public Health & Policy, London School of Hygiene & Tropical Medicine, Keppel Street, London, WC1E 7HT UK

## Abstract

**Background:**

Early pregnancy loss (unintended pregnancy loss before 20 completed weeks of gestation) is a common adverse pregnancy outcome, with previous evidence reporting incidence ranging from 10 to 30% of detected pregnancies. The objective of this systematic review and meta-analysis is to determine the incidence and range of early pregnancy loss in contemporary pregnant populations based on studies with good internal and external validity. Findings may be useful for clinical counseling in pre-conception and family planning settings and for people who experience early pregnancy loss.

**Methods:**

We will search MEDLINE, EMBASE, and CINAHL databases using combinations of medical subject headings and keywords. Peer-reviewed, full-text original research articles that meet the following criteria will be included: (1) human study; (2) study designs: controlled clinical trials or observational studies with at least 100 pregnancies in the denominator, or systematic reviews of studies using these designs; (3) conducted in high-income countries; (4) reporting early pregnancy loss incidence, defined as unintended early pregnancy loss occurring prior to 20 weeks’ gestation expressed as the number of losses among all pregnancies in the study period; (5) among a contemporary (1990 or later) general population of pregnancies; and (6) published between January 1, 1990, and August 31, 2021. We will assess the quality of included studies according to the United States Preventive Services Task Force Criteria for Assessing Internal and External Validity of Individual Studies. If appropriate, based on methodological comparability across included studies, we will conduct meta-analyses using random effects models to estimate the pooled incidence of early pregnancy loss among all studies with both good internal and external validity, with meta-analyses stratified by study design type (survey-based or self-reported and medical record-based), by induced abortion restrictions (restricted vs. unrestricted), and by gestational age (first trimester only vs. all gestational ages before 20 weeks).

**Discussion:**

This systematic review will synthesize existing evidence to calculate a current estimate of early pregnancy loss incidence and variability in reported incidence estimates in high-income settings. The findings of this review may inform updates to clinical counseling in pre-conception and family planning settings, as well as for patients experiencing early pregnancy loss.

**Systematic review registration:**

We have registered this review with the International Prospective Register of Systematic Reviews (PROSPERO #226267).

**Supplementary Information:**

The online version contains supplementary material available at 10.1186/s13643-021-01815-1.

## Background

Early pregnancy loss (unintended pregnancy loss before 20 completed weeks of gestation) is a common adverse pregnancy outcome, with reported incidence ranging from 10 to 30% of detected pregnancies [1–3]. Early pregnancy loss spans a range of subtypes, including spontaneous abortion, missed abortion, incomplete abortion, molar pregnancy, and others. Current clinical practice guidelines on early pregnancy loss diagnosis and management published by the American College of Obstetricians and Gynecologists (ACOG) and the UK’s National Institute for Health and Care Excellence (NICE) cite incidence estimates of 10% [4] and 20% [5], respectively [2, 6, 7]. The estimates are drawn from small (*n* < 600), highly selected clinical cohort studies conducted in the 1980s–1990s [1–3]. These estimates may not be applicable to general pregnant populations due to the selection of predominantly white and college-educated pregnant people with a planned pregnancy. With many changes in the characteristics of pregnant populations in the past 30 years (particularly increased frequency of delayed childbearing [8], assisted reproductive technology utilization [9], and pregnancies to persons with comorbidities such as obesity, diabetes mellitus, or hypertension [10–12]), the incidence of early pregnancy loss reported in studies before 1990 may not be generalizable to contemporary populations.

Furthermore, these commonly cited estimates of early pregnancy loss incidence may not be applicable to clinical counseling in most settings. This is because these estimates are based on the detection of pregnancy and early pregnancy loss within studies with specific research protocols [1, 3] that are unlikely to be replicated in standard pregnancy experience and care outside of research settings. These studies collected prospective, serial beta-human chorionic gonadotropin measurements to identify pregnancies and losses. Outside of research settings, pregnancy identification is based on pregnancy symptoms or pregnancy tests conducted at home or at a health care visit at approximately 5–6 weeks of gestation, with 23–28% of pregnancies being identified ≥7 weeks’ gestation. For clinical counseling purposes, determining the incidence of early pregnancy loss among pregnancies identified in non-research settings is more relevant because this reflects the risk for standard patients [13, 14].

Estimates of early pregnancy loss among pregnancies identified in non-research settings can be obtained from several sources, including population-based administrative databases (e.g., pregnancy registries created by abstracting from medical records and/or billing claims data based on clinical pregnancy care) and reproductive health surveys (e.g., routinely administered nationally representative reproductive health surveys such as the National Survey of Family Growth [15], from the USA, or the National Survey of Sexual Attitudes and Lifestyles [16] from the UK). Population-based administrative database studies have yielded early pregnancy loss incidence estimates ranging from 12% [17] to 17% [18], while surveys have yielded estimates from 18% [19] to 24% [10]. These population-based estimates may better approximate clinically relevant measures of early pregnancy loss incidence than those drawn from highly selected clinical cohorts.

The objective of this systematic review is to determine the incidence and range of early pregnancy loss in contemporary pregnant populations based on studies with good internal and external validity. Findings may be useful for clinical counseling in pre-conception and family planning settings and for people experiencing early pregnancy loss [20–22].

## Methods

The design and implementation of this systematic review will adhere to the Preferred Reporting Items for Systematic Review and Meta-Analysis Protocols (PRISMA-P) 2015 statement [23]. We have registered this review with the International Prospective Register of Systematic Reviews (PROSPERO) (systematic review identification number 226267). This study was deemed exempt from ethics review from the University of British Columbia and Children’s and Women’s Health Centre of British Columbia Research Ethics Board.

The key question underlying this systematic review is: “What is the incidence of early pregnancy loss in contemporary general pregnant populations in high-income settings?” We will approach this question using the modified PICOT framework [24] outlined in Table 1.

**Table 1:** Modified PICOT chart outlining the framework underlying this systematic review of the literature reporting early pregnancy loss incidence

	Included	Excluded
**Population**	All recognized pregnancies, defined by either self-report or report to a health care provider or indicated by a pregnancy-related clinical encounter, in OECD-classified high-income settings, representing general pregnant populations.	Pregnancies not recognized by the pregnant person or pregnancies occurring in a setting not classified as high-income by OECD; study populations restricted to assisted reproductive technology settings
**Outcome**	Early pregnancy loss, defined as unintended pregnancy loss occurring prior to 20 weeks’ gestation expressed as the number of losses among all pregnancies in the study period, including all types of unintended pregnancy loss occurring before 20 weeks of gestation or only common types of early pregnancy loss (spontaneous, missed, incomplete abortions only)	Induced abortion, stillbirth (intrauterine fetal demise after 20 completed weeks of gestation), and less common subtypes of early pregnancy loss examined separately (hydatidiform mole/molar pregnancy, anembryonic pregnancy, ectopic pregnancy)
**Time**	Jan 1, 1990–August 31, 2021	Before Jan 1, 1990, or after August 31, 2021

Because this systematic review is not evaluating a specific intervention or exposure, our PICOT framework does not include an intervention or comparator components

*OECD* Organization for Economic Cooperation and Development

### Data sources and search strategy

We will search MEDLINE, EMBASE, and CINAHL databases, using a search strategy developed in consultation with a research librarian (see Additional file 1: Search Strategy) using relevant medical subject headings and keywords. We piloted the search strategy for each database using a list of 17 papers that we identified a priori as meeting our intended inclusion criteria; we revised our search strategy to minimize the number of abstracts that would need to be reviewed while capturing these key papers in our search (Table 2). We will augment this search strategy by hand-searching the bibliographies of eligible studies meeting inclusion criteria for additional relevant articles not identified in our database search and a preliminary gray literature key-word search using Google (for relevant newspaper articles, retracted publications, and other publications from relevant stakeholders) and gray literature databases (OpenGrey, TRIP Pro, New York Academy of Medicine’s Grey Literature Report, and Canadian Institute for Health Information). We will review and evaluate gray literature using the same criteria as the peer-reviewed literature.

**Table 2:** Search strategy summary for a systematic review of early pregnancy loss in high-income settings

Keywords	
	Early pregnancy loss*, spontaneous abortion*, miscarriage*, fetal loss*, pregnancy loss*, anembryonic pregnanc*, embryonic loss*, spontaneous miscarriage*, early pregnancy failure*, pregnancy failure*, hydatidiform mole, septic abortion*, missed abortion*, incomplete abortion*, ectopic pregnanc*, risk*, rate*, prevalen*, inciden*, report*, trend*, impact*Keywords terms for high-income countries (as defined by World Bank; “High-Income OECD Countries”^25^) (see Additional file 1: Search Strategy).
Database-specific subject headings	
*MEDLINE*	*Subject heading (MeSH):* Spontaneous abortion, septic abortion, missed abortion, incomplete abortion, ectopic pregnancy, , hydatidiform mole, risk, morbidity, incidence, prevalence. MeSH terms for high-income countries (as defined by World Bank; “High Income OECD Countries”^25^) (see Additional file 1: Search Strategy).
*CINAHL*	*Subject heading:* Spontaneous abortion, incomplete abortion, hydatidiform mole, ectopic pregnancy, incidence, prevalence, morbidity.
*EMBASE*	*Subject heading (Emtree):* Spontaneous abortion, septic abortion, incomplete abortion, missed abortion, blighted ovum, hydatidiform mole, risk, morbidity, incidence, prevalence. Emtree terms for high-income countries (as defined by World Bank; “High-Income OECD Countries”^25^) (see Additional file 1: Search Strategy).

### Eligibility criteria

Peer-reviewed, full-text original research articles that meet all of the following inclusion criteria will be included in this review: (1) human study; (2) study designs: controlled clinical trials or observational studies (prospective cohort, retrospective cohort, cross-sectional, chart review, survey) with at least 100 pregnancies in the denominator, or systematic reviews of studies using these designs; (3) conducted in high-income countries (as defined by the World Bank; “High-Income OECD Countries” [25]); (4) reporting early pregnancy loss incidence, defined as unintended early pregnancy loss occurring prior to 20 weeks’ gestation expressed as the number of losses identified in typical practice settings (i.e., not specific research settings) among all pregnancies in the study period; (5) among a contemporary (1990 or later) general population of pregnancies; (6) published between January 1, 1990, and August 31, 2021. Studies meeting at least one of the following exclusion criteria will be excluded: (1) non-human study; (2) controlled clinical trials or observational studies with fewer than 100 pregnancies in the denominator, studies using any other designs (case-control, case reports or series, editorials, opinions, commentaries, or other designs not specified in inclusion criteria); (3) conducted outside OECD high-income setting; (4) not reporting early pregnancy loss incidence; (5) study population not contemporary (including pregnancies exclusively before January 1, 1990); (6) study population restricted to assisted reproductive technology setting; (7) study population sampled using any sampling method (e.g., restriction, oversampling, case-control) based on a clinical condition or characteristic, defined as any condition, disease, or characteristic with a diagnosis code, treatment with a specific medication or procedure, or recruitment from a specialty clinic serving those with a specific disease or condition. Studies that selected the study population based on age, body mass index, or other social, demographic, or environmental characteristics will not be excluded under this criterion; (8) studies reporting only uncommon types of early pregnancy loss (ectopic pregnancy, molar pregnancy, hydatidiform mole, anembryonic pregnancy); and (9) published before January 1, 1990, or after August 31, 2021.

To prevent publication bias arising from restriction to studies published in English [26], we will include studies published in languages other than English using translation services at our University or Google Translate.

In settings where induced abortion services were restricted by law or strict regulations, induced abortions may be erroneously reported as early pregnancy loss [27]. Because the legal status and availability of induced abortion services vary by setting (even within OECD high-income countries), we will categorize included studies based on whether induced abortion access was “restricted” vs. “unrestricted” using classifications published yearly by the Centre for Reproductive Rights [28] (see Additional file 2: Induced abortion regulation classification). This will allow us to examine whether reported early pregnancy loss incidence varies by induced abortion regulatory status (by examining incidence separately among studies in settings with restricted and unrestricted induced abortion access at the time of study data collection). In the event that reported incidence estimates do vary meaningfully, we will conduct a quantitative bias analysis to account for misclassification of induced abortions as early pregnancy losses [29].

### Study selection and data management

Two reviewers (NO, MO) will independently dual-screen titles and abstracts for eligibility and will review full texts of studies that were screened and determined to not meet at least one exclusion criterion. Any discrepancies will be discussed until consensus is reached. A third reviewer (LS, doctoral-level reproductive epidemiologist) will also review a random selection of 50 abstracts against inclusion/exclusion criteria and will discuss with the two primary reviewers, provide any training required, adjudicate to resolve any discrepancies for the remaining abstracts, and oversee the full-text reviews. We will import all eligible articles from the primary search into an Endnote library, then to the Covidence platform for systematic reviews [30]. We will remove duplicates using Covidence. The first reason for exclusion will be documented in Covidence based on our PICOT framework using this hierarchy: (1) population not meeting our eligibility criteria, (2) outcome definition not meeting our eligibility criteria, or (3) time outside our eligibility window.

The two reviewers will extract study details and findings using standardized data extraction forms (see Additional file 3: Data Extraction Form). Extracted data will include author and year published, study setting (including induced abortion restriction classification) [31], study period, study design, sample size, early pregnancy loss incidence (numerator *n*, denominator *n*, percentage, and standard error), specific early pregnancy loss type(s) studied with definitions, subgroups examined, exposure(s)/risk factors examined, and gestational age range and distribution for early pregnancy losses. See Table 3 for characteristics to be extracted.

**Table 3:**
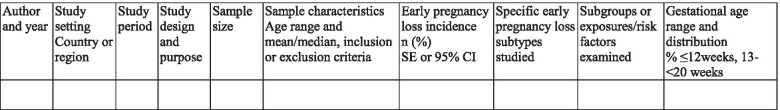
Characteristics of included studies reporting early pregnancy loss incidence in high-income settings

#### Quality assessment

Two reviewers (NO and MO) will independently evaluate and rate the quality of the included studies according to the United States Preventive Services Task Force, Criteria for Assessing Internal and External Validity of Individual Studies [32]. To finalize the quality assessment ratings, the two reviewers and senior reviewer (LS) will meet to discuss until consensus is reached on the quality rating for internal and external validity for all included studies. We will rate the internal validity of included studies as “good,” “fair,” or “poor” based on these criteria. We will evaluate internal validity according to information bias (studies must clearly and appropriately define early pregnancy loss and total pregnancies; studies that only include inpatient hospitalization data will have a maximum rating of “fair”) and selection bias (study sample should be representative of those eligible for inclusion, or the study base; loss to follow-up should be less than 20% for a “good” internal validity rating). Since our review will examine reported estimates of early pregnancy loss, but not associations with specified exposures, the importance of confounding bias is minimal in this review. For a study to be rated as having “good” internal validity, it must meet all study design-specific criteria, as laid out by United States Preventive Services Task Force. If a study misses at least one criterion, it will be rated “fair.” If a study has important limitations or a fatal methodological flaw, it will be rated “poor.” External validity assessment is rated as “poor,” “fair,” or “good” and will focus on the generalizability of findings to underlying regional or national populations. Our assessment of external validity will consider the similarity of the regional or national population being represented to the general obstetric population of high-income settings (the target population for this review). For example, a maximum external validity rating of “good” would require the study population to be the full population or employ representative sampling from the full population of pregnancies in an OECD high-income country or large region (e.g., state or province, multiple counties or cities); restriction to one city or county would result in a maximum external validity rating of “fair”; restriction to one hospital/clinic, region, or sample characteristic (e.g., occupation, racial/ethnic group) would result in a maximum external validity rating of “poor”. Likewise, a maximum external validity rating of “good” requires that the age range use the full reproductive age range (or at least ages 18–44), while restriction to an age range of more than 10 years would result in an external validity rating of “fair,” and restriction to an age range <10 years would result in a rating of “poor.” External validity ratings will also consider pregnancy detection methods that do not apply in standard clinical or at-home pregnancy detection settings. If a study population is restricted by marital status (e.g., only married people), pregnancy intention (e.g., only planned pregnancies), or pregnancy history (e.g., parity or loss), the maximum external validity rating will be “fair.” For survey studies, we will report response rates, which are an important component of external validity for this study design. See Table 4 for a drafted table showing reported incidence and quality assessments. See Additional file 4: Quality Assessment Rationale for criteria that will be used in rating study internal and external validity.

**Table 4:**
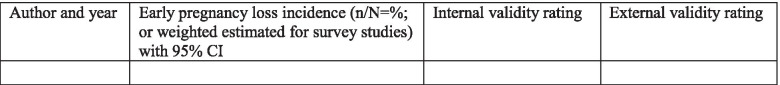
Incidence of early pregnancy loss and quality assessment for studies included in the systemic review

#### Meta-analysis

If deemed appropriate, based on methodological comparability across included studies, we will conduct a quantitative meta-analysis of early pregnancy loss incidence to combine estimates from all studies with good internal and external validity. We will present incidence estimates from individual studies included in the meta-analysis along with the pooled incidence from the meta-analysis (Table 5). We will conduct the meta-analysis using random effects models with inverse-variance weighting using the Hartung-Knapp-Sidik-Jonkman method to estimate an average incidence value for early pregnancy loss across studies; this method is robust to biases that can arise due to a small number of included studies or variability in sample size across studies [33]. We will assess heterogeneity among included studies according to (i) overlap of confidence intervals around estimated incidence values, (ii) the *I*^2^ statistic (a quantification of inconsistency among studies, indicating the proportion of variability across studies that is due to heterogeneity), (iii) and the Tau^2^ statistic (*τ*^2^, a measure of between-study variance). We will consider an *I*^2^ value < 25% to indicate non-important heterogeneity, 25–<50% to indicate modest heterogeneity, 50–<75% to indicate substantial heterogeneity, and ≥75% to indicate considerable heterogeneity [34]. In addition, we will calculate a 90% prediction interval around our overall early pregnancy loss incidence estimate, which provides a measure of dispersion or variability in incidence values from the underlying populations from which study cohorts were drawn [35].

**Table 5:**
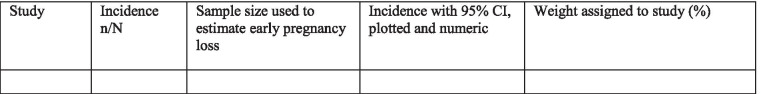
Incidence of early pregnancy loss in high-income settings, meta-analysis findings

We will conduct meta-analyses stratified by gestational age (first trimester only vs. all gestational ages before 20 weeks) and study design type (administrative data derived from medical records vs. survey or patient-reported data) and by induced abortion restrictions (restricted vs. unrestricted). We will examine heterogeneity within strata of the stratified analysis to determine if accounting for the clinical and methodological sources of heterogeneity that we identified a priori are sufficient to account for any important observed heterogeneity. We will conduct all meta-analyses using Stata 14.0.

## Discussion

This systematic review will examine the incidence of early pregnancy loss among contemporary populations in high-income settings. This review will provide the best available evidence regarding early pregnancy loss incidence and the range of incidence values. We will determine variability in reported incidence by study design, induced abortion legal status, and gestational age for early pregnancy losses and summarize these findings using meta-analysis.

Findings of this systematic review and meta-analysis will need to be interpreted in light of potential limitations. Early pregnancy loss includes a range of subtypes, which may be etiologically heterogeneous. By combining these subtypes, this review will not determine the incidence of each type of early loss separately and will specifically exclude studies focused only on rare subtypes. The expected data sources include data extracted from medical records (e.g., using health administrative data) and self-reported data (e.g., from survey studies), which can generate estimates subject to early pregnancy loss misclassification. Although our bias analysis will specifically account for induced abortions being misclassified as pregnancy losses, there may be further misclassification (e.g., stillbirths occurring after 20 weeks of gestation misclassified as an early pregnancy loss), which we will not account for analytically.

Pre-conception and family planning clinical counseling, as well as clinical care for patients experiencing early pregnancy loss, should include current information on the best available estimates of early pregnancy loss risk. Given that current obstetrics clinical practice guidelines related to early pregnancy loss care report incidence based on highly selected populations published 20–30 years ago, clinical counseling may not reflect the best available evidence on this incidence of this common adverse pregnancy outcome.

## Supplementary Information

**Additional file 1.** Search Strategy.

**Additional file 2.** Induced abortion regulation classification table.

**Additional file 3.** Data extraction form.

**Additional file 4.** Quality Assessment Rationale.

## Data Availability

All data included in this systematic review and meta-analysis will be drawn from published original research and will be available through those papers directly. We will make our meta-analysis dataset publicly available along with the completed meta-analysis final paper.
