# Supplementary material for: Early pregnancy loss incidence in high-income settings: a protocol for a systematic review and meta-analysis
Source: Syst Rev. 2021 Oct 25;10:274. doi: 10.1186/s13643-021-01815-1 (PMC8543941; doi:10.1186/s13643-021-01815-1)
Supplement: Supplementary file 1 — Additional file 1. Search Strategy. [file 13643_2021_1815_MOESM1_ESM.docx]

**Additional File 1: Search Strategy**

High Income OECD Countries:

Antigua; Aruba; Australia; Bahamas; Bahrain; Barbados; Barbuda; Bermuda; Brunei; Canada; Cayman; Chile; China; Croatia; Curacao; Cyprus; Czech Republic; Denmark; Finland; France; French Polynesia; German; Gibraltar; Greece; Greenland; Guam; Hong Kong; Hungary; Iceland; Ireland; Israel; Italy; Japan; Korea; Kuwait; Liechtenstein; Luxembourg; Macau; Malta; Monaco; Netherlands; New Caledonia; Northern Mariana Islands; New Zealand; Norway; Oman; Palau; Panama; Poland; Portugal; Puerto Rico; Qatar; San Marino; Saudi Arabia; Scotland; Seychelles; Singapore; Slovakia; Slovenia; Spain; St. Kitts; Sweden; Switzerland; Taiwan; Trinidad and Tobago; Turks and Caicos; United Arab Emirates; United Kingdom; United States; Uruguay; Virgin Islands

Database: Ovid MEDLINE(R) and Epub Ahead of Print, In-Process, In-Data-Review & Other Non-Indexed Citations, Daily and Versions(R) <1946 to August 31, 2021>

Search Strategy:

--------------------------------------------------------------------------------

1 ((Early pregnancy loss* or spontaneous abortion* or miscarriage* or fetal loss* or pregnancy loss* or anembryonic pregnanc* or embryonic loss* or spontaneous miscarriage* or early pregnancy failure* or pregnancy failure* or hydatidiform mole or septic abortion* or missed abortion* or incomplete abortion* or ectopic pregnanc*) adj10 (risk* or rate* or prevalen* or inciden* or report* or trend* or impact*)).ti,ab,kf. (15811)

2 morbidity/ or exp incidence/ or exp prevalence/ (587907)

3 exp Risk/ (1271334)

4 exp spontaneous abortion/ or exp hydatidiform mole/ or exp septic abortion/ or exp missed abortion/ or exp incomplete abortion/ or exp ectopic pregnancy/ (54236)

5 2 or 3 (1646268)

6 4 and 5 (6325)

7 1 or 6 (19355)

8 exp Canada/ (165540)

9 (Canad$ or "Nova Scotia$" or "New Brunswick$" or Newfoundland$ or "Labrador$ Prince Edward Island$" or Quebec$ or Ontari$ or Manitoba$ or Saskatchewan$ or Alberta$ or "British Columbia$" or Yukon$ or "Northwest Territori$" or Nunavu$).ti,ab,kf. (177631)

10 exp United States/ (1387339)

11 ("United States" or US or America$ or Alabam$ or Alaska$ or Arizona$ or Arkansas$ or California$ or Colorad$ or Connecticut$ or Delaware$ or Florid$ or Georgia$ or Hawaii or Idaho$ or Illinois$ or Indiana$ or Hoosier or Iowa$ or Kansa$ or Kentuck$ or Louisiana$ or Maine$ or Maryland$ or Massachusetts$ or Michigan$ or Minnesota$ or Mississippi$ or Missouri$ or Montana$ or Nebraska$ or Nevada$ or "New Hampshir$" or "New Jersey$" or "New Mexic$" or "New York$" or "North Carolin$" or "North Dakota$" or Ohio$ or Oklahoma$ or Oregon$ or Pennsylvania$ or "Rhode Island" or "South Carolina" or "South Dakota" or Tennessee or Texas or Utah or Vermont or Virginia$ or Washington$ or Wisconsin$ or Wyoming$).ti,ab,kf. (1510298)

12 exp Australia/ (152442)

13 (Australia$ or "New South Wales" or "New South Welsh" or "Northern Territor$" or Queensland$ or Tasmania$ or Victoria$).ti,ab,kf. (167377)

14 andorra/ or austria/ or belgium/ or exp baltic states/ or croatia/ or czech republic/ or hungary/ or poland/ or slovakia/ or slovenia/ or france/ or germany/ or gibraltar/ or greece/ or ireland/ or italy/ or liechtenstein/ or luxembourg/ or cyprus/ or malta/ or monaco/ or netherlands/ or portugal/ or san marino/ or spain/ or switzerland/ (668104)

15 (Andorra$ or Austria$ or Belgi$ or Estonia$ or Latvia$ or Lithuania$ or Croatia$ or Czech or Hungar$ or Poland or Polish or Slovak$ or Slovenia$ or France or French or German$ or Gibraltar or Greece or Greek or Ireland or Irish or Italy or Italian or Liechtenstein$ or Luxembourg$ or Malta or Maltese or Cyprus or Cypriot or Monaco or Monacan or Monegasque or Netherlands or Dutch or Portug$ or "San Marino" or Sammarinese or Spain or Spanish or Switzerland or Swiss).ti,ab,kf. (757752)

16 "scandinavian and nordic countries"/ or denmark/ or greenland/ or finland/ or iceland/ or norway/ or sweden/ (208261)

17 (Scandinavia$ or Denmark or Danish or Faroe$ or Greenland$ or Finland or Finnish or Iceland$ or Norway or Norwegian or Sweden or Swedish).ti,ab,kf. (214142)

18 united kingdom/ or channel islands/ or england/ or northern ireland/ or scotland/ or wales/ (356618)

19 ("United Kingdom" or British or Britain or "Channel Island$" or England or English or Scotland or Scottish or Wales or Welsh or "Isle of Man" or Manx).ti,ab,kf. (301031)

20 "antigua and barbuda"/ or bahamas/ or barbados/ or british virgin islands/ or puerto rico/ or "saint kitts and nevis"/ or "trinidad and tobago"/ or united states virgin islands/ (9952)

21 (Antigu$ or Barbud$ or Baham$ or Barbad$ or "Virgin Island$" or "Puerto Ric$ St. Kitts" or "Saint Kitts" or Nevis$ or Kittitian or Trinidad$ or Tobago$ or Cayman$ or "Turks and Caicos").ti,ab,kf. (6897)

22 aruba/ or caribbean netherlands/ or curacao/ or sint maarten/ (47)

23 (Aruba$ or Curacao$ or "Saint-Martin$" or "Saint Martin$" or "St. Martin$" or "Sint Maarten$").ti,ab,kf. (886)

24 Bermuda/ (191)

25 Bermud$.ti,ab,kf. (1665)

26 Panama/ (2470)

27 Panama$.ti,ab,kf. (4693)

28 chile/ or uruguay/ (16456)

29 (Chile$ or Uruguay$).ti,ab,kf. (22087)

30 hong kong/ or macau/ or japan/ or "republic of korea"/ or taiwan/ (227256)

31 ("Hong Kong$" or Cantonese or Macau or Macao or Macanese or Japan$ or "South Korea$" or "Republic of Korea" or Taiwan$).ti,ab,kf. (314836)

32 brunei/ or singapore/ (14356)

33 (Brunei$ or Singapore$).ti,ab,kf. (17958)

34 new caledonia/ or guam/ or palau/ or new zealand/ (42884)

35 ("French Polynesia$" or Guam$ or Palau$ or "New Caledonia$" or "New Zealand$" or "Northern Mariana$").ti,ab,kf. (61689)

36 bahrain/ or israel/ or kuwait/ or oman/ or qatar/ or saudi arabia/ or united arab emirates/ (53187)

37 (Bahrain$ or Israel$ or Kuwait$ or Oman$ or Qatar$ or Saudi$ or "United Arab Emirates" or Emirati$ or Emirian$ or Emiri).ti,ab,kf. (68792)

38 Seychelles/ (396)

39 Seychell$.ti,ab,kf. (838)

40 8 or 9 or 10 or 11 or 12 or 13 or 14 or 15 or 16 or 17 or 18 or 19 or 20 or 21 or 22 or 23 or 24 or 25 or 26 or 27 or 28 or 29 or 30 or 31 or 32 or 33 or 34 or 35 or 36 or 37 or 38 or 39 (4979617)

41 7 and 40 (5117)

***************************
