# Supplementary material for: Early pregnancy loss incidence in high-income settings: a protocol for a systematic review and meta-analysis
Source: Syst Rev. 2021 Oct 25;10:274. doi: 10.1186/s13643-021-01815-1 (PMC8543941; doi:10.1186/s13643-021-01815-1)
Supplement: Supplementary file 2 — Additional file 2. Induced abortion regulation classification table. [file 13643_2021_1815_MOESM2_ESM.docx]

**Additional File 2: Induced abortion regulation classification table**

| **Country** | Abortion restriction classification |
| --- | --- |
| Antigua | 1 |
| Aruba | 1 |
| Australia | 0 |
| Bahamas | 1 |
| Bahrain | 1 |
| Barbados | 1 |
| Barbuda | 1 |
| Bermuda | 1 |
| Brunei | 1 |
| Canada | 0 |
| Cayman | 1 |
| Chile | 1 |
| China | 0 |
| Croatia | 0 |
| Curacao | 0 |
| Cyprus | 0 |
| Czech Republic | 0 |
| Denmark | 0 |
| Finland | 1 |
| France | 0 |
| French Polynesia | 0 |
| Germany | 0 |
| Gibraltar | 1 |
| Greece | 0 |
| Greenland | 0 |
| Hong Kong | 0 |
| Hungary | 0 |
| Iceland | 0 |
| Ireland | 0 |
| Israel | 1 |
| Italy | 0 |
| Japan | 1 |
| Korea | 0 |
| Kuwait | 1 |
| Liechtenstein | 1 |
| Luxembourg | 0 |
| Macau | 0 |
| Malta | 2 |
| Monaco | 1 |
| Netherlands | 0 |
| New Caledonia | 0 |
| Northern Mariana Islands | 0 |
| New Zealand | 0 |
| Norway | 0 |
| Oman | 1 |
| Palau | 2 |
| Panama | 1 |
| Poland | 1 |
| Portugal | 0 |
| Puerto Rico | 0 |
| Qatar | 1 |
| San Marino | 2 |
| Saudi Arabia | 1 |
| Scotland | 0 |
| Seychelles | 1 |
| Singapore | 0 |
| Slovakia | 0 |
| Slovenia | 0 |
| Spain | 0 |
| St. Kitts | 1 |
| Sweden | 0 |
| Switzerland | 0 |
| Taiwan | 1 |
| Trinidad and Tobago | 1 |
| United Arab Emirates | 1 |
| United Kingdom | 1 |
| United States | 0 |
| Uruguay | 0 |
| Virgin Islands | 0 |
| 0, abortion available on request (gestational limits may vary)  1, abortion available with specific health, social, or economic grounds  2, abortion prohibited altogether  ^1^ The World’s Abortion Laws. Centre for Reproductive Rights. Accessed September 10, 2021. Available from: <https://maps.reproductiverights.org/worldabortionlaws> | |
