# Supplementary material for: Early pregnancy loss incidence in high-income settings: a protocol for a systematic review and meta-analysis
Source: Syst Rev. 2021 Oct 25;10:274. doi: 10.1186/s13643-021-01815-1 (PMC8543941; doi:10.1186/s13643-021-01815-1)
Supplement: Supplementary file 3 — Additional file 3. Data extraction form. [file 13643_2021_1815_MOESM3_ESM.docx]

**Additional File 3: Data extraction form**

General Information:

- First author and Publication Year
- Study setting (country, state/province, city/hospital) and Study period
- Journal title & citation

Study Information:

- Study design and data source (i.e. randomized control trial, prospective cohort, retrospective cohort, cross-sectional, chart review)
- Study population inclusion/ exclusion criteria
- Pregnant female age distribution: (mean/standard deviation and/or % <20, ≥35)
- Gestational age distribution mean/ standard deviation and/or %≤12weeks, ≥13 weeks, any other information provided)
- Description of population (descriptors in table 1)
- Early pregnancy loss definition used
- Early pregnancy loss subtypes examined
- Exposures/risk factors examined and any reported association with Early pregnancy loss incidence
- Subgroups examined, variation in incidence by subgroup

Outcomes:

- n (number of early pregnancy loss events)
- N (sample size, denominator)
- Reported incidence, with standard error and/or 95% confidence intervals [Note to use weighted incidence/ standard error or confidence intervals for survey studies and indicate with * that estimate is weighted]

Quality Assessment including rationale:

- US Preventive Services Task Force External Validity rating (poor, fair, good)
- US Preventive Services Task Force Internal Validity rating (poor, fair, good)

Notes and Additional Data:

- Strengths
- Limitations (described in paper + our assessment)
- Key conclusions
