# Supplementary material for: Early pregnancy loss incidence in high-income settings: a protocol for a systematic review and meta-analysis
Source: Syst Rev. 2021 Oct 25;10:274. doi: 10.1186/s13643-021-01815-1 (PMC8543941; doi:10.1186/s13643-021-01815-1)
Supplement: Supplementary file 4 — Additional file 4. Quality Assessment Rationale. [file 13643_2021_1815_MOESM4_ESM.docx]

**Additional File 4: Quality Assessment Rationale**

Internal validity rationale

- Information bias
  - *Strengths*
    - Good: Addresses information bias through use of hospital records *and* self-reported measures (i.e., within-study validation)
    - Good: early pregnancy loss measure/ascertainment method is based on a previously validated approach
  - *Weaknesses*
    - Fair: early pregnancy loss detected from (inpatient) hospitalization data only (expect substantial under-ascertainment)
- Selection bias
  - *Strengths*
    - Good: No loss to follow-up
    - Good: >80% complete follow-up
  - *Weaknesses*
    - Fair: 50-79% complete follow-up
    - Poor: <50% complete follow-up

External validity rationale

- Age range
  - *Strengths*
    - Good: all ages (no restrictions) or at least includes the age range 18-44
  - *Weaknesses*
    - Does not capture full reproductive age range
      - Poor: age range <10 years
      - Fair: age range ≥10 years
- Sample population
  - *Strengths*
    - Good: Study population is comprised of full population of pregnancies in geographic area of interest
    - Good: Study population is representative of full population of pregnancies in geographic area of interest
    - Study population is not restricted by marital status
    - Study population includes planned and unplanned pregnancies
    - Study population not selected on the basis of fertility history and/or reproductive outcome
  - *Weaknesses, geographic*
    - Fair: Study population is restricted to one city/region
    - Poor: Study population is restricted to one hospital/clinic, unless paper reports a broad hospital catchment area more similar to that of a region
  - *Weaknesses, demographic*
    - Fair: Study population is restricted by characteristic (occupation, racial/ethnic group)
  - *Weaknesses, fertility/reproductive history*
    - Poor: Study population was selected on the basis of fertility history and/or reproductive outcome (e.g. previous birth(s)/loss(es), etc.)
  - *Weaknesses, marital status or pregnancy intention*
    - Fair: Study population is restricted to married couples
    - Fair: Study population does not include unplanned pregnancies
- **Pregnancy detection**
  - *Strengths*
    - Good: Pregnancy and early pregnancy loss detection methods are applicable for clinical/at home pregnancy detection
      - E.g. human chorionic gonadotropin test at home or doctor’s office, onset of menstruation
  - *Weaknesses*
    - Fair: Pregnancy and early pregnancy loss detection methods are not applicable for standard clinical/at home pregnancy detection
      - E.g. research protocols with daily urine samples
